# Case of Cystotomia

**Published:** 1845-10-01

**Authors:** James P. White


### Case of Cystotomia;

*Rendered necessary by lacerations of the Urethra in using a Catheter: High operation; Recovery.*

Communicated for the Buffalo Medical Journal, by JAMES P. WHITE, M. D.

On the 30th of April last, at 11 P. M., I was called to visit Dr. M., an elderly gentleman of full habit, who, as he informed me, had been a good deal troubled in passing his water for several years past. He had ridden during the afternoon a distance of thirty miles over a rough road, and been frequently obliged to stop and urinate. These efforts were attended with great pain, and a small quantity of urine only passing at each effort.—I found him in a warm hip bath, with a vessel before him containing a gill, or more, of coagula and bloody water. I also learned that he had been in the habit of using a gum elastic Catheter, frequently, himself; that he had not been able to introduce it at that time, although he had used "considerable force, and it would almost pass." That undue force had been resorted to was but too clearly manifested by its effects. On asking for the Catheter used, I found it was old, much roughened at the end, torn at the fenestra and containing an unusually stiff stylet. On passing my hand over the hypogastric region, I could not discover any distension of the bladder, and was convinced the patient had mistaken the difficulty, and was treating himself for retention whilst suffering with strangury, and more or less inflammatory action in the prostate and neck of the bladder. At his urgent solicitation I introduced the Catheter, and found it considerably impeded in the prostatic region, which was now exceedingly tender, and I had no doubt, had been severely lacerated in his rude efforts to thrust the instrument forward. By following closely under the arch, I was enabled, with very slight effort, to pass a medium sized, silver catheter completely into the bladder. A small quantity of turbid, offensive urine followed, and the stimulus of the catheter in that viscus excited violent and spasmodic efforts to urinate. The Doctor was by this time satisfied to submit to appropriate treatment. The pulse being full and frequent, tongue parched and reddened, and pain intense, I drew 30 ℥ of blood from the arm, and directed ℞ Opii pulv. grs. vi.
Tart. Ant. et Potass. grs. iss.
Prot. Chl. Hydrarg. grs. x. M ft. pulv. iii.
one to be taken every 2 hours; flax seed tea for drink, and foment the bowels with warm hops and vinegar.

May 1st, 5 A. M., has taken all the powders: has not been able to sleep, pain constantly returning, with paroxysmal efforts to urinate, and severe beyond endurance. Directed a half pint of flax seed tea containing ii ʒ tinct. opii to be thrown into the rectum, to be repeated in an hour, if necessary to procure relief; fomentations to be continued, and the following ℞ Tart. Ant. et Potass. grs. ii. dissolve in 12 table spoons of water, and take one every two hours. 8 A. M. pain somewhat lessened; pulse has risen to 112, full and hard; bowels slightly tender in hypogastric region; has not passed any urine since the introduction of catheter at 12 last night. Repeated the bleeding to ℥ xxxii., and gave Ol. Ricini ℥ ii. 11 A. M. has been more quiet since the last bleeding, but still suffers considerable pain in pubic and lumbar regions. Directed 12 leeches to be applied to lower part of abdomen, to be followed by warm fomentations; continue the solution and repeat the oil at 12 o'clock. 2 P. M., more comfortable; pulse slower and tongue moist. 6 and 10 P. M. no evacuations from bowels, although oil had been repeated three times in increased doses; still, the patient was more comfortable, and bowels were not perceptibly distended. Directed large injections of warm water to be thrown up the rectum at 12 and 3, with a small quantity of spirits turpentine added.

May 2d, 7 A. M., has slept a little during the night; bowels have not moved, nor has he passed any urine; can distinguish the bladder, but not yet greatly distended.

℞ Fol. Sennae ʒ ii.

Sulph. Magnesiæ ℥ ii. M. and infuse, and take one third every 2 hours unless the bowels move. Cupped the sacrum freely with great relief to the pain in that region. Made slight efforts to introduce the catheter, but immediately desisted in consequence of the pain occasioned. It might also be stated that the point of the syringe in giving the enemas, gave great pain, and an examination per rectum indicated a tender and inflamed state of perineum and prostate, with enlargement of the latter. Repeated the hip bath. 12, he has vomited the senna tea, and most, if not all the oil taken yesterday. 3 P. M., bowels tender over hypogastrium, and bladder a good deal distended; pain increased, ℞ Opii pulv. grs. xii.
Prot. chlor. Hydg. xxiv. M. ft. pil vi.

one to be taken every 2 hours, if necessary to relieve the pain and gastric irritability. During the succeeding few hours, made occasional, gentle efforts to introduce the catheter, but was arrested about one inch from the bladder, at the same point where the lacerations were suspected on the night of my first visit. At 6 P. M., and from that time to 9 or 10 o'clock, pain in consequence of distension was almost insupportable.—During this time Prof. Hamilton visited the patient in connection with myself, and also made several unsuccessful efforts to introduce catheters of various forms and sizes, being uniformly arrested at the same point. At 12 we resorted to considerable force, but did not succeed in penetrating the bladder, although we used a firm silver catheter, and it seemed carried sufficiently far, and in the right direction.

May 3d 2 A. M., the distension was so great that it was deemed, in consultation with Drs. Trowbridge and Hamilton, unsafe longer to delay opening the bladder. He had now been about 60 hours without passing half a gill of urine. The whole perineal space, and the prostate being exquisitely tender, and the latter much enlarged, the danger of fistulous opening before the inflammation in that region should so far abate as to permit the flow of urine through the urethra, and the great difficulty to be apprehended in retaining any instrument in the wound if made thro' the rectum, induced us to adopt the high operation. I accordingly commenced an incision three inches above the symphysis pubis, and continued it downward about two inches, dividing the integuments and adipose tissue, [which was very thick] to the linea alba. Into the lower part of this wound I introduced a long curved trochar, and canula. As soon as I felt it completely within the bladder, the trochar was withdrawn with one hand, whilst, with the other, the canula was carried backward and downward 6 or 7 inches to the basfond. We were unable to ascertain the exact quantity of urine which escaped, one of the vessels containing it being emptied by inadvertence. It was, however, estimated at nearly four pints. The patient immediately experienced great relief, and even fell asleep during the adjustment of the dressings.

The canula was retained by means of tapes passing around the body, and attached to the instrument with a silk cord or ligature in front. Into this was inserted a small, long, gum-elastic tube, which, by being very much flexed down to the side, was, with a little suction, converted into a syphon. This soft tube was passed completely through the canula, and, as was supposed, flexed under it; by so doing not only was the bladder more completely evacuated, but the irritation which might arise from the pressure of the metallic instrument upon the mucous membrane prevented. The wound was filled with lint dipped in warm water, and over that, fitted neatly to the stem of the canula, was spread a piece of oiled silk.—The patient was left in charge of an intelligent assistant, who was directed frequently to apply the mouth to the exposed end of the syphon, to make sure that it did not become obstructed,and infiltration ensue. 8 A. M. 6 hours after the operation, patient has slept quietly most of the time; urine has been discharged drop by drop as it was secreted; bowels have moved freely during the time, being probably prevented from so doing previously, by the mechanical pressure of the distended bladder upon the rectum. Pulse frequent and soft, tongue moist, and febrile symptoms greatly diminished. Continued the anodyne after the following form:

℞ S. Moph. grs. ii.
Aqua Destil. ℥i. M.

Take a tea-spoon-full every 4 hours; diet to consist chiefly of flax seed tea and other mucilages.

May 4th 8 A. M., passed a comfortable night; has slept most of the time during the last 30 hours; bowels have moved several times, and are considerably distended; urine continues to flow freely through the syphon in the canula; pulse 96 and soft; thirst not urgent. Continued the anodyne in diminished doses; made his drinks more nutritious, and gave chalk mixture to check hypercatharsis, and relieve acidity of stomach. Removed gum elastic tube, inserted a new one, and again passed it beyond the lower end of the metallic canula, that it may coil under it and protect the membrane from injurious pressure. 9 P. M. continues comfortable.

May 5th, 8½ A. M., is doing well. Diet slightly improved, and permitted him to be moved with great care from one bed to another. Evening, continues comfortable.

May 6th, morning, nothing unusual has occurred, seems doing well. Evening, wound has a slightly offensive odor, and directed it to be dressed with sol. chlor. sodæ. He has also a slight increase of febrile symptoms.

May 7th, the patient is doing well; wound looks well, offensive smell has disappeared.

May 8th, sat up some time and was very comfortable. A bag of oiled silk is attached to the external orifice of the canula as a substitute for the syphon, which became impervious.

May 9th 10 A. M., seems doing well. In dressing the wound a slight rotation of the body retracted the canula an inch or two; finding it difficult of reinstation, left it entirely out, and after making slight, but unsuccessful efforts to insert a long probe and a small gum catheter, concluded to wait until the bladder should become more distended, when, unless the urine could be readily passed per urethra, the distension would favor the reintroduction of instruments. Besides, I did not apprehend danger from extravasation, believing that before this period the tissues were entirely adherent, forming a perfect canal. 8 P. M. still comfortable; bladder not much or painfully distended; introduced a long probe through the fistula into the bladder but could not insert the canula, and left in the probe.

May 10, 2 A. M., pain in hypogastric region considerable; could not pass his urine by the urethra. It was remarkable to see how little ulcerative action had taken place in the lips of the wound in the bladder, although a metallic instrument had been kept constantly in the wound during the last seven days. The small probe seemed fitted with perfect exactness. Although considerable lateral pressure was made, as with a lever, and the bladder was full, not a drop of urine escaped. We now fastened a cord to the eye of the probe which was exposed, then passed the long end of the cord through the canula, and one held the probe by the string to prevent its pressure upon the posterior walls of the bladder, whilst the other, not without some force, carried the canula forward, being directed by the probe into the bladder. The discharge of about a pint of urine gave immediate relief, and the patient slept well during the remainder of the night.

May 10, 9 A. M., the patient is comfortable, and the canula is more perfectly secured and retained in the fistula, discharging the water as it is secreted.

From this time to the 18th, the patient allowed the urine to flow guttatim. At this time the discharge of bloody, purulent matter, consequent upon the lacerations of the urethra, having nearly ceased, we commenced corking the mouth of the canula, and discharging it at intervals, in the hope that slight distension might enable him to pass the urine by the urethra.

June 5th. Since the last date the patient's general health continued to improve. Leeches were occasionally applied to the perineum until all pain and irritation had subsided in the prostatic region, and the urethra seemed free from disease. The bladder, meantime, retained the urine from five to seven hours without difficulty, when it was drawn off, at first, by unstopping the mouth of the canula, and finally, this was removed, and a gum elastic catheter inserted through the fistula from time to time, at his convenience. On this day, for the first time, the introduction of the catheter through the urethra was attempted. A medium sized, highly curved, silver catheter was selected, and after a few gentle efforts, it slipped into the bladder. A small quantity of muco-purulent matter escaped with the urine.

June 11th, the patient has thus far been unable to evacuate his urine by urethra, although he retains it the usual length of time, and draws it off readily by means of the catheter, through the fistulous opening. Strength much improved; rides out, and lives comfortably well. Whether the bladder has lost its contractile power by over distension, or is fastened to the anterior walls of the abdomen at the point of incision by adhesions, it is difficult to determine. The facility with which the catheter was introduced, as well as the subsidence of all irritation in the prostatic region, render it improbable that its flow is longer prevented by pressure of the prostate upon the urethra.

June 12th, the patient had two slight urinary evacuations per vias naturales, which he attributes to increase of strength. He was obliged to resort to the catheter and the fistula after he returned from a long walk fatigued. Directed him to drink freely of an infusion of uva ursi and buchu.

June 25th, the urethral and vesical irritation seems within the last few days to have been translated to the right testicle and its envelopes, which, notwithstanding the use of leeches, fomentations, and other appropriate discutients, suppurated, and was opened on the 30th. The discharge was copious, and the diseased action seemed entirely confined to the cellular and cutaneous tissues.

July 10th, the patient has continued daily to improve since the last date; passes his urine through the urethra without difficulty, although, as a measure of precaution, he continues to introduce the catheter through the fistulous opening occasionally, to prevent its closing entirely. He is able to walk and ride, his appetite and strength in a great measure restored, and he leaves to-day for his residence, distant some forty-five miles.

Sept. 15th, Dr. M. being in town called to report his present condition.

His general health is unusually good. He passes his urine readily by the urethra most of the time, but has continued to pass a gum elastic catheter through the sinus every day to prevent entire adhesion. He states that "urinary difficulties have not been greater during the last two months than during the same space of time before the operation." He has resorted to the sinus on two or three occasions instead of passing the catheter as heretofore, when any irritation or obstruction occurred, through the urethra. The sympathetic action excited in the testicle has entirely subsided, leaving no doubt that the diseased action was confined to the skin and cellular tissue. By my direction he has procured a silver tube about 3½ inches in length, slightly enlarged or bulbous at one end, and with a button head at the other end, which may be entirely closed with a cork. The sinus retains this short catheter without irritation, and by being able to evacuate the bladder, whenever it is necessary to resort to artificial means, through this tube, it is hoped the irritation so long existing in the neck of the bladder and prostate will subside, and a permanent cure be effected of a malady of many years standing.

*Buffalo, September*, 1845.

